# Secondary loss of a *cis-*spliced intron during the divergence of *Giardia intestinalis* assemblages

**DOI:** 10.1186/1756-0500-7-413

**Published:** 2014-06-30

**Authors:** Ryoma Kamikawa, Yuji Inagaki, Tetsuo Hashimoto

**Affiliations:** 1 Graduate School of Human and Environmental Studies, Kyoto University, Yoshida-Nihonmatsu cho, Kyoto 606-8501, Japan; 2 Graduate School of Global Environmental Studies, Kyoto University, Kyoto, Japan; 3 Graduate School of Life and Environmental Sciences, University of Tsukuba, Tsukuba, Ibaraki, Japan; 4 Centre for Computational Sciences, University of Tsukuba, Tsukuba, Ibaraki, Japan

**Keywords:** Intron loss, Homologous recombination, Reduced genome, Reverse transcription

## Abstract

**Background:**

*Giardia intestinalis* is a parasitic unicellular eukaryote with a highly reduced genome, in which only six *cis-*spliced and four *trans-*spliced introns have been discovered. However, we anticipate that more *cis-* and *trans-*spliced introns likely remain unidentified in genes encoding hypothetical proteins that occupy ca. 2/3 of all of the open reading frames (ORFs) in the *Giardia* genome. Consequently, comprehensive surveys of introns in ORFs for hypothetical proteins are critical for better understanding of the intron evolution in this organism.

**Results:**

In this study, we identified two novel *cis*-spliced introns in the draft genome data of *G. intestinalis* strain WB, by surveying the conserved sequence motifs shared amongst the previously known introns. *G. intestinalis* strains can be divided into phylogenetically distinct assemblages A–H, and all the introns identified in past studies are shared among the published genome data from strains WB, DH, GS, and P15 representing assemblages A1, A2, B, and E, respectively. Nevertheless one of the two novel introns identified in this study was found to be absent in strain P15.

**Conclusion:**

By considering the organismal relationship among *G. intestinalis* assemblages A1, A2, B, and E, one of the two introns identified in this study has highly likely been lost after the divergence of the assemblages. On the basis of a sequence comparison between the intron-bearing loci in WB, DH, and GS genomes and the homologous but intron-free locus in P15 genome, we propose that the loss of this particular intron was mediated by integration of the DNA fragment reverse-transcribed from mature mRNAs.

## Background

Spliceosomal introns, which are excised from pre-mature mRNAs by RNA-protein complexes called spliceosomes [1], are one of the features exclusively found in eukaryotic genomes. However, a large variety in intron density has been found across eukaryotic genomes sequenced to date [2]. In the human genome, for example, 8.4 introns on average are annotated per gene [3], and the mean intron size is ca. 3,000 bp in length [2]. In contrast, *Giardia intestinalis*, a unicellular eukaryotic parasite belonging to the Diplomonadida (Excavata) is known to possess a highly reduced genome of only 12 Mbp in length [4]. One of the prominent natures of the *Giardia* genome is its low intron density—only 6 *cis*-spliced introns and 4 *trans*-spliced introns (split introns) have been identified prior to this study [4-12]. Henceforth here, we simply designate *cis*-spliced introns as ‘introns,’ and *trans*-spliced introns as ‘splintrons’ [8].

Most of introns/splintrons in the *Giardia* genome were identified principally as non-coding stretches intervening in open reading frames (ORFs) encoding proteins shared amongst phylogenetically diverse eukaryotes. However, the simple procedure described above may be problematic for distinguishing the coding and non-coding regions (i.e. exons and introns/splintrons) in functionally unidentified ORFs encoding *Giardia*-specific proteins. Since unidentified ORFs occupy approximately 2/3 of the ca. 9,000 ORFs encoded in the *Giardia* genome [4], a large fraction of introns/splintrons in the *Giardia* genome may have been overlooked by pioneering surveys principally based on sequence similarity.

To shed light on introns/splintrons veiled in unidentified ORFs in the genome of *G. intestinalis* strain WB, we conducted an intron survey based on the conserved sequence motifs in introns/splintrons, and successfully detected two novel introns in unidentified ORFs (Note that our approach is not technically applicable to survey splintrons). The two ORFs, which harbor introns in the WB genome, were identified in the genomes of *G. intestinalis* strains DH [13], GS [14] and P15 [15] as well, but one of these in the P15 genome were found to be intron-free. We propose a scenario to explain the presence/absence of the particular intron in the four *G. intestinalis* strains.

## Methods

### *In silico* detection of conserved intron sequences

*Giardia* introns/splintrons known to date bear conserved sequence motifs at the 5′ and 3′ termini, 5′-STATG-3′ and 5′-HCTRACMCVCAG-3′ (R = A or G; H = A, T, or C; M = A or C; V = A, C, or G; S = G or C), respectively. Furthermore, the two motifs may be flanked with each other within 300 bp, since all of the known introns in the *Giardia* genome range from 29 to 220 bp in length. We searched for genome segments that satisfied the above criteria in the draft genome data of *G. intestinalis* strain WB (GiardiaDB, http://www.giardiadb.org/giardiadb/).

### Cells, DNA, RNA, and reverse transcription

*G. intestinalis* strain WB (ATCC50803) was cultivated as described previously [7]. Genomic DNA (gDNA) was extracted by cetyl trimethylammonium bromide buffer [16] from the harvested cells. Total RNA was isolated from the cells with the RNeasy Plant Mini kit (QIAGEN) following the manufacturer’s instruction. To synthesize cDNA from total RNA, reverse transcription was performed by the 3′ rapid amplification of cDNA ends kit (Invitrogen) following the manufacturer’s instruction.

### Detection of intron splicing

We designed exact-match primers at the 5′ and 3′ flanking regions of intron-like sequences nominated by the *in silico* survey (see above), and performed two separate PCRs, one with total gDNA as the template (gDNA-based PCR) and the other with cDNA as the template (cDNA-based PCR). If the particular candidate is an intron, the amplicons from cDNA-based PCR should be shorter than those from gDNA-based PCR by the intron length. We examined all intron-like sequences by comparing size difference between gDNA-based and cDNA-based PCR amplicons. In addition, we sequenced the cDNA-based PCR amplicons to assess whether intron-like sequences were excised. The experimental procedures above identified that two intron-like sequences, one in ORF no. AACB02000068-1-10039-10248 and the other in ORF no. AACB02000001-6-305427-304747, were excised *in vivo* (see below). Sets of primers 5′-GAAAAAAAATCCAGAGATGGC-3′ and 5′-TTGCAAAGTGCAATGAAAGC-3′, and 5′-AAACAGGTTCGTCAATATCAC-3′ and 5′-AGGATACGAAGCGTTGCGAA-3′ were used for examining the former and latter introns, respectively. Amplified PCR products were cloned into the pGEM T-easy vector (Promega) and sequenced completely.

We experimentally determined the 5′ ends of the mRNAs from ORFs AACB02000068-1-10039-10248 and AACB02000001-6-305427-304747 by using the 5′ rapid amplification of cDNA ends kit (Invitrogen) following the manufacturer’s instruction. Cloning and sequencing were performed as described above.

## Results and discussion

**Figure 1:**
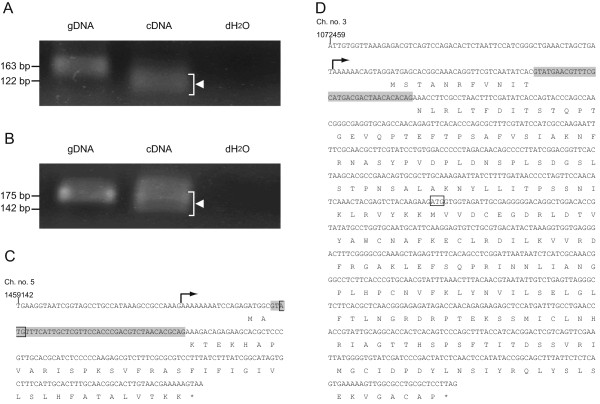
**Novel *cis*-spliced introns in *Giardia intestinalis *strain WB (assemblage A). A**. Evidence for *cis*-splicing of the intron in the ORF AACB02000068-1-10039-10248 (orfA). PCR products amplified from gDNA, cDNA, and distilled water were applied to the left, middle, and right lanes, respectively. The expected lengths of the amplicons from gDNA and cDNA are 163 and 122 bp, respectively. The amplicon marked by an arrowhead was cloned and sequenced. **B**. Evidence for *cis*-splicing of the intron in the ORF AACB02000001-6-305427-304747 (orfB). Details are described in A. The expected lengths of the amplicons from gDNA and cDNA are 175 and 142 bp, respectively. **C**. Intron position in the ORF AACB02000068-1-10039-10248 (orfA). Nucleotide sequence from positions 1,459,142 to 1,459,363 of chromosome no. 5 is shown. The initiation codon of the originally annotated hypothetical protein GL50803_37070 is enclosed by an open square. The intron region is shaded. The 5′ terminus of the transcript (arrow) was determined experimentally. The revised amino acid sequence is provided as one-letter code. **D**. Intron position in the ORF AACB02000001-6-305427-304747 (orfB). Nucleotide sequence from positions 1,071,651 to 1,072,459 of chromosome no. 3 is shown. The initiation codon of the originally annotated hypothetical protein GL50803_15525 is enclosed by an open square. Other details are same as described in **C**.

We identified two novel introns among the 11 intron-like sequences nominated by the *in silico* survey of introns in the genome data of *G. intestinalis* strain WB. For the two novel introns, the intron-free transcripts (i.e. mature mRNAs) were successfully identified in the amplicons from cDNA-based PCR (Figure 1A and B). Curiously, we detected both intron-bearing and intron-free transcripts (i.e. pre-mature and mature mRNAs) in the cDNA-based amplicons that were not clearly distinguished in size from the gDNA-based amplicons (Figure 1B), suggesting that the splicing efficiency of this particular intron is relatively low. The above conjecture about the splicing efficiency is consistent with the fact that five out of the eight clones for the cDNA-based amplicons were found to contain the intron (data not shown). For the rest of the intron candidates, we failed to show any experimental evidence for splicing (data not shown).

The two introns were found in ORFs no. AACB02000068-1-10039-10248 and AACB02000001-6-305427-304747, shown in Figure 1C and D, respectively. Hereafter, we designate the ORFs AACB02000068-1-10039-10248 and AACB02000001-6-305427-304747 as orfA and orfB, respectively. Each of the novel introns locates at the 5′ terminal region of the corresponding ORF, as seen in the previously identified *Giardia* introns, except for that found at the 3′ terminal region of rpl7A gene. In terms of intron length, both the intron in orfA (41 bp) and that in orfB (33 bp) are comparable with other *Giardia* introns (29–36 bp), except for the intron in the ORF encoding hypothetical protein GL50803_35332 (220 bp) and that in rpl7A gene (109 bp). Based on the experimentally confirmed 5′ ends of the mRNAs, the gene models for the two ORFs, as well as their intron-exon boundaries, were refined (Figure 1C and D).

**Figure 2:**
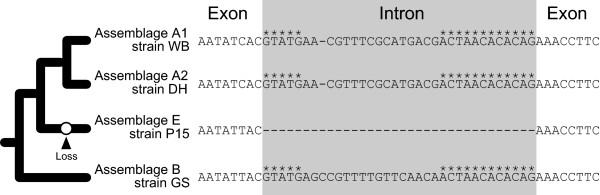
**Secondary loss of an intron in *Giardia intestinalis *assemblage E represented by strain P15.** The relationship among *G. intestinalis* assemblages A1, A2, B, and E based on Monis et al. [19,20] is schematically provided on left. On right, we compare the intron and its flanking exon sequences of the ORF AACB02000001-6-305427-304747 (orfB) in strain WS (assemblage A1) and the homologous regions in the ORF AHGT01000002-2-45140-45820 in strain DH (assemblage A2), the ORF ACVC01000101-5-40937-40224 in strain P15 (assemblage E), and the ORF ACGJ01002919-1-19018-19737 in strain GS (assemblage B). Intron regions are shaded. The conserved sequence motifs shared amongst *Giardia* introns/splintrons are highlighted by asterisks. Gaps are represented by dashes. We here propose that (i) the intron found in orfB in strain WS (this study) can be traced back to the common ancestor of the four assemblages, and then (ii) a secondary loss of the intron occurred after assemblages A1/A2 and E separated from each other (highlighted by open circle).

The strains of *G. intestinalis* can be divided into 9 assemblages (A1, A2, B–H) in light of their sequence diversity [4,17,18]. All of the introns/splintrons identified in past and present studies, except for that in orfB (see below), were found to be shared across the draft genome data of the four *G. intestinalis* strains WB, DH, P15, and GS, which represent assemblages A1, A2, E, and B, respectively. Unlike other introns/splintrons, the orfB intron may not be ubiquitously present in *G. intestinalis* strains (Additional file 1: Figure S1A and B)—an intron-bearing orfB was found in the GS genome (ORF ACGJ01002919-1-19018-19737) and in the DH genome (ORF AHGT01000002-2-45140-45820), but we identified only the intron-free homologue in the P15 genome (ORF ACVC01000101-5-40937-40224). It is believed that assemblages A1 and A2 are the closest relatives to each other, and A1/A2 complex is more closely related to E than B [19,20]. Thus, we propose that (i) the common ancestor of the four assemblages possessed an intron-containing orfB, and (ii) orfB in assemblage E lost the corresponding intron after the split of assemblages A1/A2 and E (Figure 2) if the corresponding locus of the published P15 genome is not derived from misassembling. To depict more detailed evolution of the intron in orfB, the genome data from *G. intestinalis* strains representing other assemblages are indispensable.

There are three major models to explain how eukaryotic genomes lost spliceosomal introns: (i) ‘de-intronization’ by mutations, (ii) non-homologous end joining (NHEJ) repair of double strand break (DSB) in an intron sequence, and (iii) homologous recombination of the cDNA e.g., [21-23]; see also Additional file 2: Figure S2A-C]. The first model assumes the conversion of an intron sequence to an exon sequence by nucleotide substitutions, which results in extension of the corresponding ORF (Additional file 2: Figure S2A). Nevertheless, the length of orfB was found to be uniform among WB, DH, GS, and P15 genomes (Figure 2; see also Additional file 1: Figure S1B), suggesting that the loss of orfB intron cannot be rationalized by deintronization. The second model demands ‘microhomology’ pairing between 5′ and 3′ splice sites to anchor the upstream and downstream exons, which are split by DSB in the intron, during NHEJ repair [22]; see also Additional file 2: Figure S2B]. Importantly, both 5′ and 3′ splice sites need to be 5′-AG/GT-3′ (the slash indicates the boundary between intron and exon) in the second intron loss model (Additional file 2: Figure S2B). As the key assumption does not fit to the splice sites of the orfB introns (Figure 2), suggesting that the intron has not been eliminated from the P15 genome by NHEJ repair. The last model invokes integration of a reverse-transcribed mRNA (i.e. cDNA; intron-free) into the original, intron-containing locus through homologous recombination (Additional file 2: Figure S2C). We regard that the homologous recombination of the cDNA, which can eliminate the entire intron sequence but does not require sequence conservation at the 5′ and 3′ splice sites, is more appropriate to explain the loss of orfB intron in the P15 genome than the two models described above, (Figure 2). It is intriguing to point out the presence of putative reverse transcriptase genes in the genome data of the four *G. intestinalis* assemblages [e.g., Genbank/EMBL/DDBJ accession nos. AF434198 (WB), AHGT01000152 (DH), EES99684 (GS), and EFO60876 (P15)], although reverse transcription activity of these encoded proteins has yet to be experimentally confirmed in *G. intestinalis* cells [24].

## Conclusion

In this study, we found two novel *cis-*spliced introns and their punctate distribution in the genomes of *G. intestinalis* assemblies. Together with the recently found *trans*-spliced introns, the data presented here suggest that the intron evolution in this organism is more complex than we previously thought.

## Competing interests

The authors declare that they have no competing interests.

## Authors’ contributions

RK determined the sequences and analyzed the data. YI and TH provided research materials. RK, YI, and TH prepared the manuscript. All the authors read and approved the final manuscript.

## Supplementary Material

Additional file 1: Figure S1 Amino acid sequences of ORFs AACB02000068-1-10039-10248 (orfA) and AACB02000001-6-305427-304747 (orfB) in *Giardia intestinalis* strain WB and the corresponding ORFs in strains GS and P15. **A**. orfA. Amino acid residues and nucleotides identical among the three sequences are shaded in black background. Asterisks indicate stop codons. The inserted position and nucleotide sequences of orfA intron are presented in the balloon. ORF nos. of orfA homologues in strains WB, DH, GS, and P15 are AACB02000068-1-10039-10248, AHGT01000085-3-18630-18839, ACGJ01001903-2-4190-4396, and ACVC01000007-3-18771-18962, respectively. **B**. orfB. The details of this figure are the same as described in A. ORF nos. of orfB homologues in strains WB, DH, GS, and P15 are AACB02000001-6-305427-304747, AHGT01000002-2-45140-45820, ACGJ01002919-1-19018-19737, and ACVC01000101-5-40937-40224, respectively. The intron sequences, which are a part of Figure 2, are not provided here. Click here for file

Additional file 2: Figure S2 Proposed models for intron loss. **A**. Deintronization by substitutions. An intron sequence (red) is changed to an exon sequence (light blue) by nucleotide substitutions (askerisks), resulting in extension of exon sequence. **B**. Non-homologous end joining repair of double strand break in intron sequence. In this model, ‘microhomology’ pairing between 5′ and 3′ splice sites anchors the upstream and downstream exons, which are split by double strand break. Subsequently, the broken strands are repaired, resulting in elimination of the entire intron sequence. **C**. Homologous recombination between an intron-containing gDNA and the corresponding intron-free cDNA (dark blue). This model assumes that the cDNA fragment, which is reverse-transcribed from a mature mRNA bearing no intron, is recombined into the corresponding intron-containing locus in the genome, resulting in elimination of the entire intron sequence. Click here for file
