# Supplementary material for: Secondary loss of a cis-spliced intron during the divergence of Giardia intestinalis assemblages
Source: BMC Res Notes. 2014 Jun 30;7:413. doi: 10.1186/1756-0500-7-413 (PMC4085374; doi:10.1186/1756-0500-7-413)
Supplement: Additional file 1: Figure S1 — Amino acid sequences of ORFs AACB02000068-1-10039-10248 (orfA) and AACB02000001-6-305427-304747 (orfB) in Giardia intestinalis strain WB and the corresponding ORFs in strains GS and P15. A. orfA. Amino acid residues and nucleotides identical among the three sequences are shaded in black background. Asterisks indicate stop codons. The inserted position and nucleotide sequences of orfA intron are presented in the balloon. ORF nos. of orfA homologues in strains WB, DH, GS, and P15 are AACB02000068-1-10039-10248, AHGT01000085-3-18630-18839, ACGJ01001903-2-4190-4396, and ACVC01000007-3-18771-18962, respectively. B. orfB. The details of this figure are the same as described in A. ORF nos. of orfB homologues in strains WB, DH, GS, and P15 are AACB02000001-6-305427-304747, AHGT01000002-2-45140-45820, ACGJ01002919-1-19018-19737, and ACVC01000101-5-40937-40224, respectively. The intron sequences, which are a part of Figure 2, are not provided here. [file 1756-0500-7-413-S1.pdf]

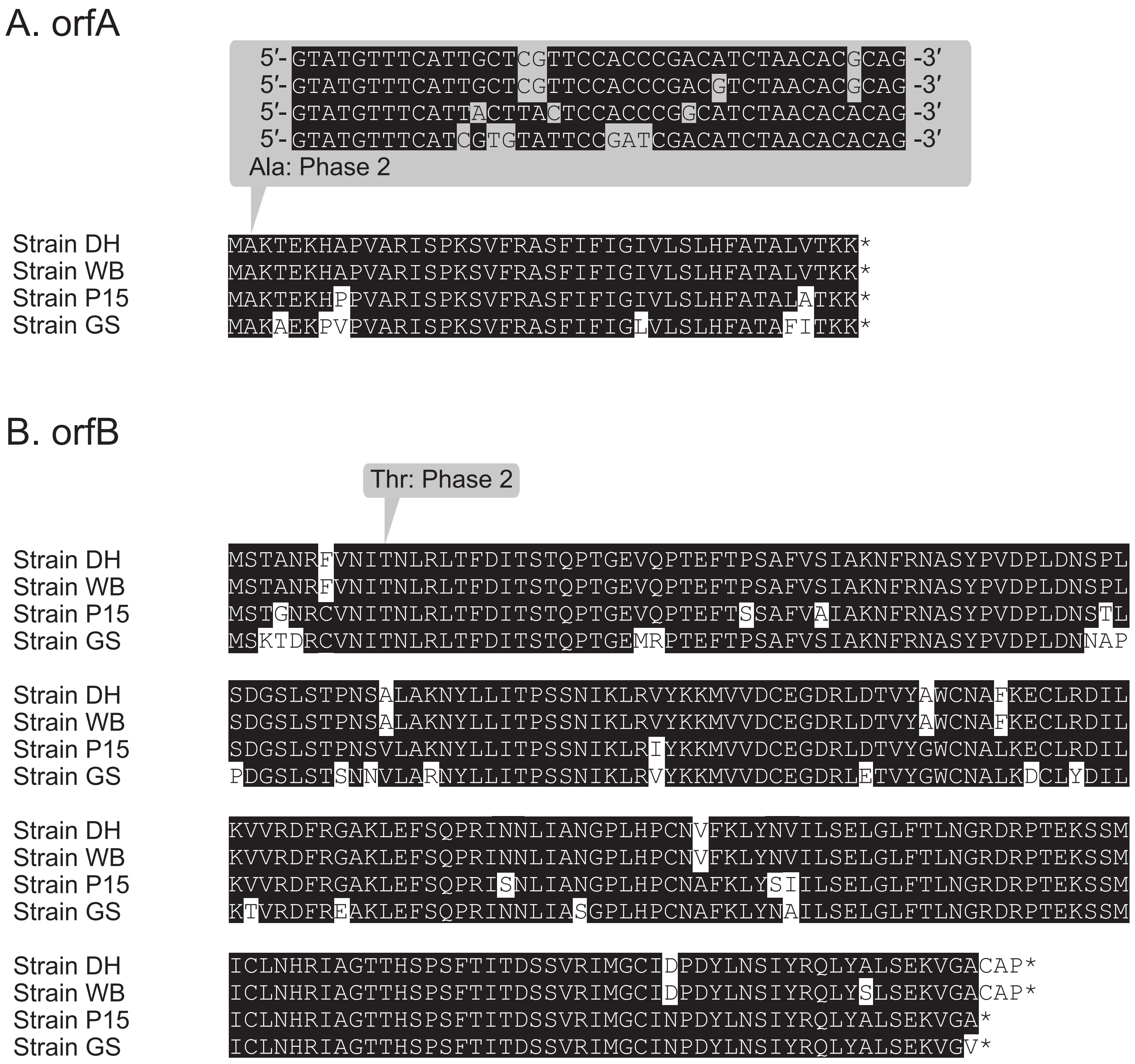

Kamikawa et al. (Supplementary Figure S1)
