# Supplementary figures and images for: Secondary loss of a cis-spliced intron during the divergence of Giardia intestinalis assemblages
Source: BMC Res Notes. 2014 Jun 30;7:413. doi: 10.1186/1756-0500-7-413 (PMC4085374; doi:10.1186/1756-0500-7-413)

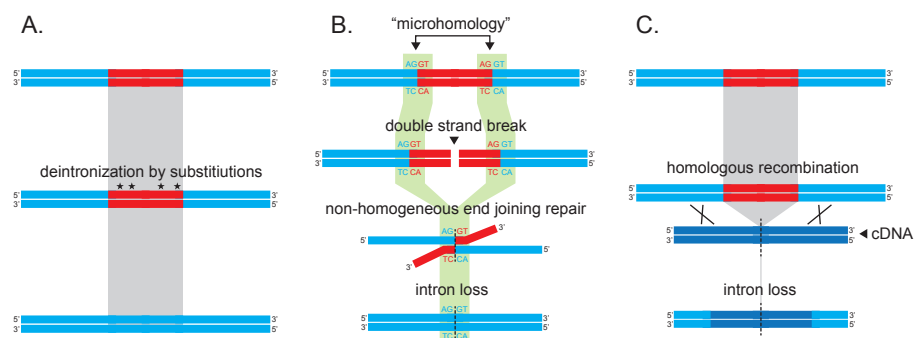

Kamikawa et al. (Fig. S2)

Supplement: Additional file 2: Figure S2 — Proposed models for intron loss. A. Deintronization by substitutions. An intron sequence (red) is changed to an exon sequence (light blue) by nucleotide substitutions (askerisks), resulting in extension of exon sequence. B. Non-homologous end joining repair of double strand break in intron sequence. In this model, ‘microhomology’ pairing between 5′ and 3′ splice sites anchors the upstream and downstream exons, which are split by double strand break. Subsequently, the broken strands are repaired, resulting in elimination of the entire intron sequence. C. Homologous recombination between an intron-containing gDNA and the corresponding intron-free cDNA (dark blue). This model assumes that the cDNA fragment, which is reverse-transcribed from a mature mRNA bearing no intron, is recombined into the corresponding intron-containing locus in the genome, resulting in elimination of the entire intron sequence. [file 1756-0500-7-413-S2.pdf]
